# Nine Years of Irrigation Cause Vegetation and Fine Root Shifts in a Water-Limited Pine Forest

**DOI:** 10.1371/journal.pone.0096321

**Published:** 2014-05-06

**Authors:** Claude Herzog, Jan Steffen, Elisabeth Graf Pannatier, Irka Hajdas, Ivano Brunner

**Affiliations:** 1 Swiss Federal Research Institute WSL, Birmensdorf, Switzerland; 2 Swiss Federal Institute of Technology Zürich ETH, Zurich, Switzerland; The Ohio State University, United States of America

## Abstract

Scots pines (*Pinus sylvestris L*.) in the inner-Alpine dry valleys of Switzerland have suffered from increased mortality during the past decades, which has been caused by longer and more frequent dry periods. In addition, a proceeding replacement of Scots pines by pubescent oaks (*Quercus pubescens* Willd.) has been observed. In 2003, an irrigation experiment was performed to track changes by reducing drought pressure on the natural pine forest. After nine years of irrigation, we observed major adaptations in the vegetation and shifts in Scots pine fine root abundance and structure. Irrigation permitted new plant species to assemble and promote canopy closure with a subsequent loss of herb and moss coverage. Fine root dry weight increased under irrigation and fine roots had a tendency to elongate. Structural composition of fine roots remained unaffected by irrigation, expressing preserved proportions of cellulose, lignin and phenolic substances. A shift to a more negative δ^13^C signal in the fine root C indicates an increased photosynthetic activity in irrigated pine trees. Using radiocarbon (^14^C) measurement, a reduced mean age of the fine roots in irrigated plots was revealed. The reason for this is either an increase in newly produced fine roots, supported by the increase in fine root biomass, or a reduced lifespan of fine roots which corresponds to an enhanced turnover rate. Overall, the responses belowground to irrigation are less conspicuous than the more rapid adaptations aboveground. Lagged and conservative adaptations of tree roots with decadal lifespans are challenging to detect, hence demanding for long-term surveys. Investigations concerning fine root turnover rate and degradation processes under a changing climate are crucial for a complete understanding of C cycling.

## Introduction

In the inner-Alpine valleys of Switzerland, increased temperatures and drought periods have become more frequent and have reached a greater extent during the last few decades [1], [2]. The most recent predictions reveal no improvement of the situation, contrariwise an increase of severe drought events is expected [3]. The most abundant tree species, Scots pine (*P. sylvestis* L.), has suffered from limited water and from increasing competition from pubescent oak (*Q. pubescens* Willd.), leading to a drastic increase in tree mortality [4], [5]. Recent publications concerned with drought induced tree mortality, explaining the interrelation of loss of hydraulic function and carbohydrate depletion, and concluding that the direct lack of water exceeds the carbon failing [6], [7]. Tree defoliation and dieback is not restricted to the Swiss alpine region. In all southern Europe forests, degeneration due to severe droughts has been recorded [8]. In a review, Anderegg et al. [9] depicted the global significance of forest dieback as a result of drought and temperature stress. Assessment of drought events are still insufficient and demand for adequate study length [10]. In addition definition of severe drought events and standardization of climate extreme studies are needed [11].

In order to reduce tree mortality, an experiment was started in 2003 with the installation of an irrigation set-up in a mature Scots pine stand in Pfynwald (Valais). After a few years of irrigation treatment, therefore reducing drought events, the forest showed explicit changes in Scots pine growth patterns. Adaptations in needle morphology, and shoot and stem growth were detected after four years of irrigation [12], as well as in the δ^13^C signal in tree rings [13].

Water uptake in trees is regulated by their roots (e.g. [14]). However, only a limited number of studies deal with fine root adaptations in forests [15]–[18]. A few studies have focused on root adaptation after irrigation combined with a fertilization treatment (e.g. [19]–[22]). In the irrigation study of the forest Pfynwald, Scots pine fine root morphology changed after four years of irrigation only slightly [23], more precisely, an increase in specific root length (SRL) [24] and a significant decrease in root tissue density (RTD) [23] were noted. In 2007, Ostonen et al. [24] reviewed the importance of SRL as a morphological parameter in fine roots to address transformations induced by changing environmental factors. Root Area Index (fine root area relative to soil area, RAI) was promoted as a useful morphological parameter to detect changes between soil types [25] or approach the effect of irrigation and fertilization on root morphology [26]. In order to detect structural changes, biochemical approaches are feasible. Several fine root compounds are known to increase with parasitic pressure [27], [28].

After death, fine roots become fine root litter. Nowadays, with global warming plant litter plays a crucial role in the carbon (C) cycle. The turnover of C in soils highly depends on the residence time of the litter. The underlying factors for litter degradation are biotic (decomposer community) and abiotic (e.g. litter quality, lignin contents, soil temperature, soil water content, pH) [29]. Litter is mostly considered to be foliar litter input on the forest floor. The fine roots of trees are an often overlooked part of litter, even though they account for 13% of the net primary production (NPP) but only account for 3% of trees biomass [30]. Fine root turnover tends to be slower than pine needle litter [31]. Importantly, it should be considered that in contrast to foliar litter, fine root litter is less exposed to weathering (e.g. water, temperature). In any case, Fujii & Takeda [32] showed that the position of the litter above or below the organic layer is of minor importance. The major factors of slow degradability of fine root litter are low water content [33] and higher lignin content [32], [34]; [29]. Rasse et al. [35] stated a faster degradation of fine root litter than shoot-derived litter in an isotope incubation experiment. The lower degradation rate is biased in nature by the physico-chemical protective properties of the soil and protection by mycorrhiza and root-hair activity. These protective mechanisms highly depend on environmental conditions such as moisture. Nevertheless, the decomposition rate of plant litter material correlates positively with nutrients but negatively with CN ratio and lignin content [36]. Hence, the question remains: can a changing environment (e.g. increased rainfall) affect the chemical structure of roots directly, or does the moisture only act as a degradation stimulant without modifying root chemical composition beforehand? The C storage potential of temperate forests therefore depends on degradability and mean lifespan of fine roots.

Natural radioactive isotope of C is a cosmogenic isotope produced in the atmosphere in reaction of thermal neutrons and ^14^N. A steady-state condition of atmospheric concentrations is maintained between production and decay. However, due to the above ground nuclear test in 1950s superficial ^14^C was produce and its concentration doubled. Presence of this so called ‘bomb peak’ global ^14^C tracer in the atmosphere has been monitored and applied during the last 50 years (for review see [37]). Broecker et al. [38] were pioneers in using this tracer to analyse C cycling in aquatic environments. Later, Trumbore [39] adapted the ^14^C approach used in the terrestrial ecosystem to analyse soil organic matter (SOM) dynamics. Gaudinski et al. [40] introduced a new application possibility for ^14^C isotope measurement to investigate mean fine root age. Further investigation and testing showed that not only new assimilated C is used for root growth but also stored C which can be older. This can lead to a mean age of C of 0.4 yr at the point of integration [41]. Several recent studies detected discrepancies in mean fine root age and postulated two pools of fine roots: one, a fast but smaller turnover pool with a mean turnover time of <1 yr, the other, larger, with a decadal turnover time [42], [43]. In many studies, fine root thickness is or was arbitrary [44]. In root age estimation and turnover studies this is not the case and is suggested for partitioning [40]. Recently, Sah and co-workers [45] tested the reliability of the radiocarbon method for determining root age whereby fine roots from ingrowth cores with a known maximum age were analyzed. Only for fine roots (<0.5 mm), the measured ^14^C age was in agreement with the ingrowth core age, thicker roots tended to be older with ^14^C measurement. Fine root age can vary largely among stands and tree species and there is a tendency for older fine root ages to be in less fertile soil [46]. Alongside increasing fine root diameters, increasing soil depth as well reveals a positive correlation with fine root age [47].

Benefiting from our nine-year irrigation study site, we attempt to fill the research gap regarding long-term adaptations of Scots pine fine root structure and composition. Is the associated vegetation of the mature pine forest affected by irrigation? Furthermore, our results will contribute to the ongoing discussion on fine root biomass increase or decrease in the topsoil after excessive water addition. Finally, is the longevity of fine roots, measured by radiocarbon dating, influenced by the water availability?

## Materials and Methods

### Site description

**Figure 1:**
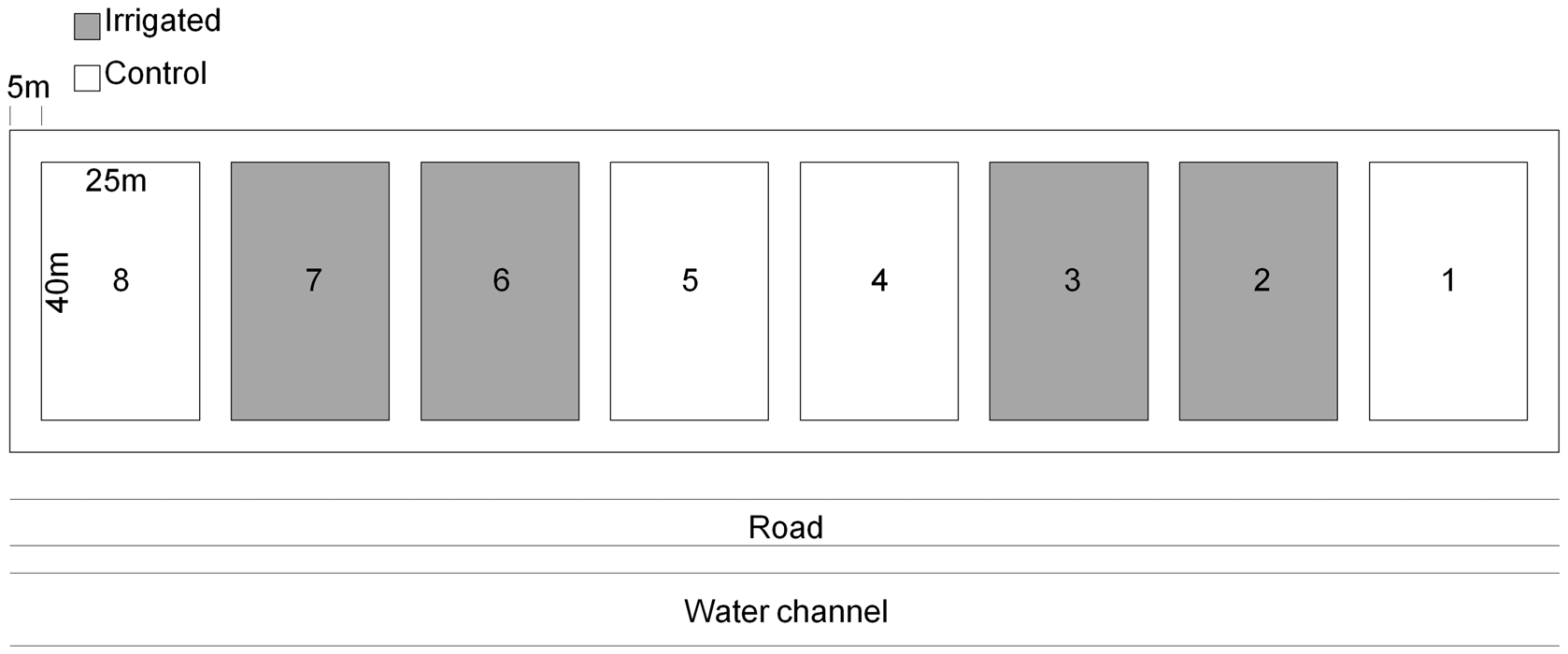
Experimental setup of the irrigation experiment in Pfynwald (VS). Irrigated plots (grey) and control plots (white) with the nearby water channel which was used for irrigation.

The irrigation experiment was situated in the Rhone Valley near Leuk (Valais, Switzerland, 46°18′ N, 7°37′ E, 615 m a.s.l.) in a Scots pine (*P. sylvestris*) forest with occasional interspersed pubescent oak (*Q. pubescens*). Permission for the field experiment was issued by the forest service of the canton Wallis (CH) (Kantonaler Forstdienst, Kreis Oberwallis, Kantonsstrasse 275, 3902 Brig-Glis). Additionally, the permission for use of the forest for research purpose was approved by the owner of the forest, the Burgerschaft Leuk (http://www.burgerschaft-leuk.ch). The geological properties are dominated by gravel input from the Rhone river and from the Illgraben alluvial cone. A more pristine pedogenic event was the landslide from Siders. The mean annual precipitation measured in Sion (20 km) was 518 mm and the mean annual temperature 10.7°C from 2003 to 2012 [48]. The irrigation experiment had 8 plots (25 × 40 m) of which four were randomly chosen for irrigation, whereas the remaining four served as control. The plots were separated by a buffer zone of 5 m (Fig. 1). From 2003 to 2012, the irrigation system was activated in rainless nights during the vegetation period (May-October), doubling the annual rainfall amount. Water from the Rhone-channel situated along the experiment site (Fig. 1) was used for irrigation. Nutrient input through irrigation was minor: phosphate was below the detection threshold (PO_4_ <0.15 kg ha^−1^ yr^−1^) and the input of nitrogen (2.4–3.3 kg ha^−1^ yr^−1^) was less than the amount that could be expected to be deposited by a doubling of rainfall (N ≤ 3.5 kg ha^−1^ yr^−1^) [49], [50]. Three identical trees per plot with the lowest crown transparency value, which refers to trees with the highest foliation, were chosen for our study [23]. In the first two plots, the volumetric soil water content was monitored hourly at a soil depth of 10 cm at four different locations using time domain reflectometry (Tektronix 1502B cable tester, Beaverton, OR).

### Vegetation

Vegetation assessment was executed to identify the vegetation shift after the nine years of irrigation. Sampling was done from 23th/24th May 2012. The coverage of vascular plant species of forbs, of shrubs and of the tree layer, were estimated in each plot using the Londo scale [51]. The nomenclature used to describe the vegetation is based on Aeschimann et al. [52]. The Landolt ecological indicator values for Swiss flora were calculated [53]. The indicator values ranged from 1 (low) to 5 (high). The nine indicators are D (aeration value), F (moisture value), H (humus value), K (continentality value), L (light value), N (nutrient value), R (reaction value (pH)), T (temperature value), W (moisture variability value). More detailed information about the indicator values is listed in Table S1.

### Fine root sampling

Fine root sampling was executed before the irrigation experiment started, on the 24^th^ April 2003 [23] and a second time after 9 years of irrigation on the 10^th^ May 2012. For the 10^th^ May 2012 sampling four soil cores at a distance of 1 m from each of the three trees per plot, were taken with an incremental borer (Ø 45 mm). The soil cores were kept refrigerated until further processing. Soil cores were washed in a sieve (mesh size 0.5 mm), and the roots were collected and separated into pine-roots, oak-roots and remaining root types. The separation of the fine roots (Ø ≤ 2 mm) was executed after following criteria: lignification (shrubs and tree roots), dichotomic branching of root tips (oak and pine only), mycorrhizal root tip size (oak < 1 mm, pine > 1 mm). This separation is merely valid for the forest site at Pfynwald. Fine roots were used for all examinations, exclusively.

### Fine root morphological and chemical analyses

The fine roots of *P. sylvestris* and *Q. pubescent* were scanned using the WinRhizo version 4.1b software package (Régent Instruments, Inc., Quebec, Canada) to obtain data regarding fine root morphology. All collected fine roots were dried (72 h, 60°C), weighted and grinded for 2 min at 80% intensity using a Retsch Mixer Mill (MM 2000, Haan, Germany).

Klason lignin of Scots pine fine roots was extracted using an adaptation of a Hiltbrunner et al. [54] protocol. Briefly described, our procedure was as follows: 200 mg of grinded fine root was weighed in falcon tubes (50 ml). Water extraction was run three times with 80°C water and 15 min incubation time per run. Centrifugation (5000 rpm) was done for 10 min. A fourth water washing step was done with ambient MilliQ water. The supernatant of the four water washing steps was then collected in order to determine the presence of phenolic substances. The washing steps were the same for the ethanol extraction. Ethanol (96%) was used at room temperature for all three rounds. Pellets were resolved in ethanol and filtered (paper filter Ø 70 mm, Schleicher Schuell, Blauband 589^3^) and dried overnight (105°C). Acid soluble lignins were extracted using H_2_SO_4_ (72%) for 1 h in a shacking bath (30°C). After having added 16.8 ml MilliQ water, the samples were autoclaved at 120°C for 1 h. Samples were filtered through porcelain filter caps (40 mL, Ø 40 mm). After weighing, the retained solid phase was burned in a muffle kiln (4 h, 550°C). The non-acid-soluble lignins were equal to the difference between the retained sample and the ash content. The acid-soluble lignins were measured with a photometric approach [55]. Therefore, the filtrate was measured at a wavelength of 205 nm in a Varian Cary 50 UV-visible spectrophotometer (Varian, USA). Overall lignin content was equal to the sum of the percentages of the dry mass acid-soluble and the non-acid-soluble lignins.

Cellulose was extracted using an adaptation of a protocol implemented by Endrulat et al. [56]. Our procedure is described here briefly: 50 mg of dried and powdered Scots pine fine roots were sealed into Teflon filter bags (F57; ANKOM Technology, Macedon, NY, USA), followed by an incubation for 2 h in 5% NaOH at 60°C to extract fats, oils, tannins and hemicelluloses, and three washing steps with deionised boiling water. To remove the lignin, a washing step at 60°C with a 7% NaClO_2_ solution for 30 h was conducted. Another three washing steps with deionised boiling water were executed before drying over night at 50°C. Using the water solubles of the lignin extraction, the phenolic substances could be quantified [57], [58]. In a 10 ml test tube, a 900-µl water extract or calibration solution was mixed with 300 µl Folin-Denis reagent (purum, Fluka, 47742) and incubated at room temperature for 3 min. 600 µl of sodium carbonate (waterfree, puriss p.a. Fluka, 71350) was added and set to rest for 3 h. With a 2 ml syringe, the 1 ml solution was filtered with a 0.2 µm syringe filter directly in a cuvette. The absorption was measured at 725 nm with a Varian Cary 50 UV spectrophotometer (Varian Com. US). δ^13^C of the extracted *P. sylvestris* fine root cellulose and bulk material was analyzed with an elemental analyser–continuous flow isotope ratio mass spectrometer (Euro-EA, Hekatech GmbH, Germany, interfaced with a Delta-V Advanced IRMS, Thermo GmbH, Germany) [56]. The ^13^C/^12^C sample values were divided by the Vienna Pee Dee Belemnite international standard (VPDB), resulting in the ratio of the ^13^C/^12^C ratio of the sample relative to the preindustrial standard VPDB (δ^13^C). C and nitrogen (N) content and CN ratios were analyzed with gas chromatography (NC-2500, Carlo Erba Instruments, Wigan, UK). Similar to the procedure used by Richter et al. [59] the extracted *P. sylvestris* fine root cellulose was combusted and graphitized [60] The ^14^C/^12^C ratio was measured using the dedicated acceleretor mass spectrometry AMS system of MICADAS at the ETH facility [61].

### Statistical analysis

All statistical analyses were executed with the open source tool R (R Development Core Team, 2011). For multivariate analyses, the add-in package VEGAN [62] and BiodiversityR [63] were required. A non-metric multidimensional scaling (NMDS, NMSrandom function) ordination technique was used for visualization of the similarity of equally treated plots. The indicator values were chosen as environmental variables to the NMDS. Their explanatory significance was tested with a permutation test comparing variables separately. A PERMANOVA (adonis function) was used for testing the variation of the vegetation in differently treated plots. To test for treatment effects, a mixed model was computed with *n* = 12 and plot as random effect by using the add-in package lme4 [64]. A likelihood ratio test was performed using the ANOVA function. Residuals were checked for normal distribution with the Kolmogorov-Smirnov test [65]. A homoscedasticity test based on Levene (1960) [66] was computed. For the statistical tests the difference between 2012 and 2003 was used (T_0_ reduction), thus reducing individual tree disparity. In case of vegetation assessment, one-way ANOVA variance analysis was executed to detect treatment-induced shifts. To estimate the age of roots based on ^14^C measurements, the data was compared to the ^14^C data measured in the atmosphere by Levin and Kromer [67] which were fitted using a polynomial regression (y = −476.5×^2^+1'222.5x−754.4, R^2^ = 0.977) (Table 1).

**Table 1:** Measured 14C and estimated fine root age before and after nine years of irrigation.

	2003			2012		
	Control	Irrigated	*p*	Control	Irrigated	*p*
14C bulk	1.14	1.15	0.591^a^	1.08	1.06	0.059
Fine root age	10.80	11.61	0.674^a^	10.42	5.66	**0.044**

a calculated from one-way ANOVA, only one sample was analyzed per plot.

14 C was analyzed with AMS system of MICADAS at the ETH facility. Age estimation using a polynomial regression (y = −476.5×^2^+1'222.5x−754.4) of atmospherical ^14^C data (Levin & Kromer 2004). Mixed model followed by ANOVA: *p*-values in bold are significant (*p* < 0.05).

## Results

### Volumetric water content in soil

**Figure 2:**
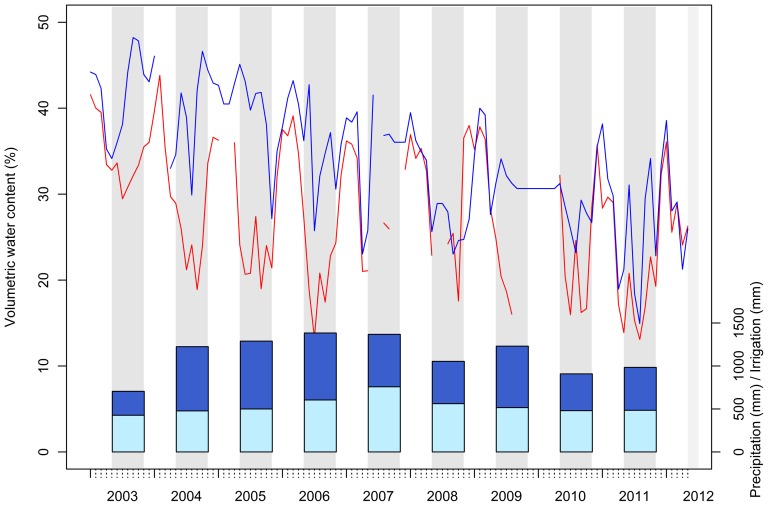
Monthly mean volumetric water content (%) of the irrigated (blue) and the control (red) plots over the experiment period (2003–2012). The annual precipitation (light blue bars) and the applied annual irrigation (blue bars) in millimetres are plotted on the second y-axis. Irrigation periods are indicated as grey bars.

The amount of water added for each irrigation period was on average 587 mm, which corresponds to a doubling of the annual precipitation amount. This resulted in a significant effect of irrigation on the volumetric water content (VWC) of the soil at 10 cm depth (*p*  =  0.022). Figure 2 illustrates the monthly mean (VWC) during the nine years of irrigation. The mean value of VWC over the nine-year period was 27.8% in the control plot and 34.3% in the irrigated plot. In some cases, the VWC were similar in both plots during failures of the irrigation system. In the wintertime and before irrigation starts in the spring, the VWC was similar in both control and irrigated plots.

### Vegetation

**Figure 3:**
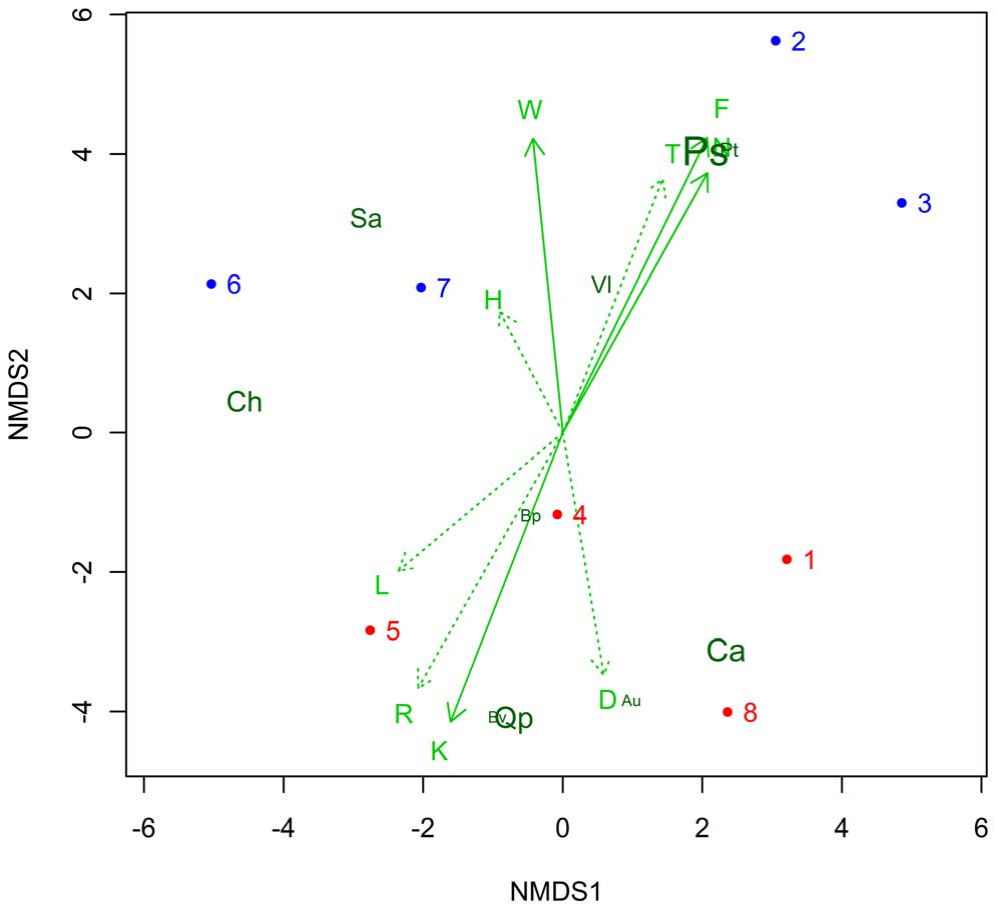
Non-metric multidimensional scaling (NMDS) of the eight plots after nine year of irrigation of the ten most abundant plant species (control = red dots, Irrigated = blue dots). The plant species are plotted with logarithmic abundance indicated by font size and orientation of their highest abundance in the plots indicated by the arrows (dark green; Ps =  *Pinus sylvestris*, Ca =  *Carex alba*, Qp =  *Quercus pubescens*, Ch =  *Carex humilis*, Sa =  *Sorbus aria*, Vl =  *Viburnum lantana*, Pt =  *Populus tremula*, Bp =  *Betula pendula*, Au =  *Arctostaphylos uva-ursi*, Bv =  *Berberis vulgaris*). The Landolt indicator values are fitted to the plot (light green arrows; significant =  solid; non-significant =  dotted) for the following properties: D (aeration value), F (moisture value), H (humus value), K (continentality value), L (light value), N (nutrient value), R (reaction value (pH)), T (temperature value), and W (moisture variability value).

The vegetation type of the pine forest belongs to the *Erico-Pinetum caricetosum albae* Br.-Bl. [68]. The species richness and the coverage rate of vegetation assessment are listed in table 2 and the full plant list and plant species mean abundance is illustrated in Table S2. Species richness did not differ between control and irrigated plots. The mean number of species in irrigated plots was 38.8 ± 1.9, whereas control plots showed a mean number of species of 37.5 ± 0.9. Coverage of vegetation differed significantly between the two treatments. Mean vegetation cover of irrigated sampling site was 50.5% ± 11.1% and 29.3% ± 9.3% in control plots. Coverage of trees showed significant differences between the two treatments. Irrigated sites showed significantly higher Scots pine coverage than controlled sites and a significant decrease of pubescent oak coverage. Coverage of dead wood, herb and moss was significantly higher in control sites than irrigated sites. Shrub cover showed no significant differences between the treatments. The calculated Landolt indicator values were also significantly higher for moisture (*p*  =  0.004), moisture variability (*p*  =  0.037) and nutrient value (*p*  =  0.041), but significantly lower for continentality (*p*  =  0.019), and the reaction value (*p*  =  0.032). These five values were significantly explanatory for the NMDS distribution of the sampling plots (Figure 3). Using a NMDS technique, the plots that experienced identical treatment showed significant clustering (PERMANOVA: *p*  =  0.023, Figure 3).

**Table 2:** Vegetation assessment results for mean number of species per plot (species richness) and mean percentage coverage of mean vegetation, dead wood, trees, shrubs, herbs, and mosses after nine years of irrigation.

	Species richness	Cover (%)					
		Mean vegetation	Dead wood	Trees	Shrubs	Herbs	Mosses
Control	37.5	29.3	9.50	55.0	16.3	28.8	50.8
Irrigated	38.8	50.5	4.50	70.0	16.3	13.8	39.5
*p*	ns	<0.001	<0.001	<0.001	ns	<0.001	<0.001

One-way ANOVA (ns = non-significant).

### Fine root morphology

Fine root morphology was altered after the nine-year irrigation period (Table 3). The dry weight per soil volume of fine roots developed in the irrigated plots increased for *P. sylvestris* and *Q. pubescens*. The remaining roots derived from other species were not affected by irrigation in their dry weight per soil volume (data not shown). Fine root tips were less frequent under irrigation, significantly for pubescent oak and slightly for Scots pine. Regarding SRL and RTD, we detected contradictory results for the two tree species. For Scots pine, SRL increased and RTD decreased whereas pubescent oak tended to react in the opposite way. Agreement in the trends between the two species was detected in the RAI. Both pine and oak increase their RAI with irrigation, though both trends are not significant.

**Table 3:** Morphological properties of *Pinus sylvestris* and *Quercus pubescens* fine roots after nine years of irrigation.

	*Pinus sylvestris*			*Quercus pubescens*		
	Control	Irrigated	*p*	Control	Irrigated	*p*
DW [mg/cm^3^]	2.66	3.92	**0.026**	0.09	0.25	**0.040**
Diameter [mm]	0.65	0.63	0.535	0.45	0.53	0.274
Root length [cm/cm^3^]	1.74	2.94	0.078	0.19	0.28	0.206
SRL [cm/mg]	0.67	0.78	0.26	2.81	1.93	0.078
RTD [mg/cm^3^]	499	451	0.306	288	351	0.236
RAI [m^2^/m^2^]	5.42	8.89	0.114	0.39	0.73	0.065
Root tips [cm^−1^]	4.39	3.73	0.063	5.19	4.06	**0.026**

Mean values are listed for fine root dry weight per soil volume (DW) and fine root morphological traits: Average diameter (diameter), root length per soil volume (root length), specific root length (SRL), root tissue density (RTD), root tips per root length (root tips). Mixed model followed by ANOVA: *p*-values in bold are significant (*p* < 0.05), (*n*  =  12).

### Fine root chemical properties

Treatment failed to induce significant differences in cellulose, phenol or lignin (Table 4). Therefore, coarse structure and the composition of the fine root chemistry persisted. There was a slight increase in cellulose under irrigation, but significant differences due to irrigation in root composition was limited to the amount of N in bulk roots. The amount of N in fine roots was reduced by irrigation. The CN ratio appeared to increase as a result of the N increase under irrigation. A highly significant result occurred in the ^13^C signal of extracted cellulose, as well as in the bulk fine root data. The ^13^C/^12^C ratio was diminished by the induced irrigation.

**Table 4:** Chemical properties of *Pinus sylvestris* fine roots before and after nine years of irrigation.

	2003			2012		
	Control	Irrigated	*p*	Control	Irrigated	*p*
Cellulose (%)	15.5	16.5	0.719	9.5	11.3	0.430
Lignin (%; *n* = 4)	37.1	37.7	0.865^a^	53.0	47.2	0.284^a^
Phenol (%)	0.17	0.17	0.933	0.18	0.18	0.983
C-bulk (%)	45.6	45.0	0.225	43.9	43.4	0.102
N-bulk (%)	0.96	1.03	0.605	0.81	0.75	**0.040**
C/N	50.5	46.9	0.519	56.1	60.5	0.080
Lignin/N (*n* = 4)	42.3	36.9	**0.040**	70.3	63.7	0.484
δ^13^C-cellulose	−25.1	−25.1	0.947	−23.9	−24.8	**0.001**
δ^13^C-bulk	−26.9	−26.8	0.773	−26.3	−27.2	**<0.001**

a calculated from one-way ANOVA, only one sample was extracted per plot.

Mean values are listed for amount of cellulose, Klason lignin, total phenol, amount of C in bulk roots (C-bulk), amount of nitrogen in bulk roots (N-bulk), the ratio of carbon to nitrogen in bulk roots (C/N) and the δ^13^C of cellulose and bulk root. Mixed model followed by ANOVA: *p*-values in bold are significant (*p* < 0.05), (*n*  =  12).

**Figure 4:**
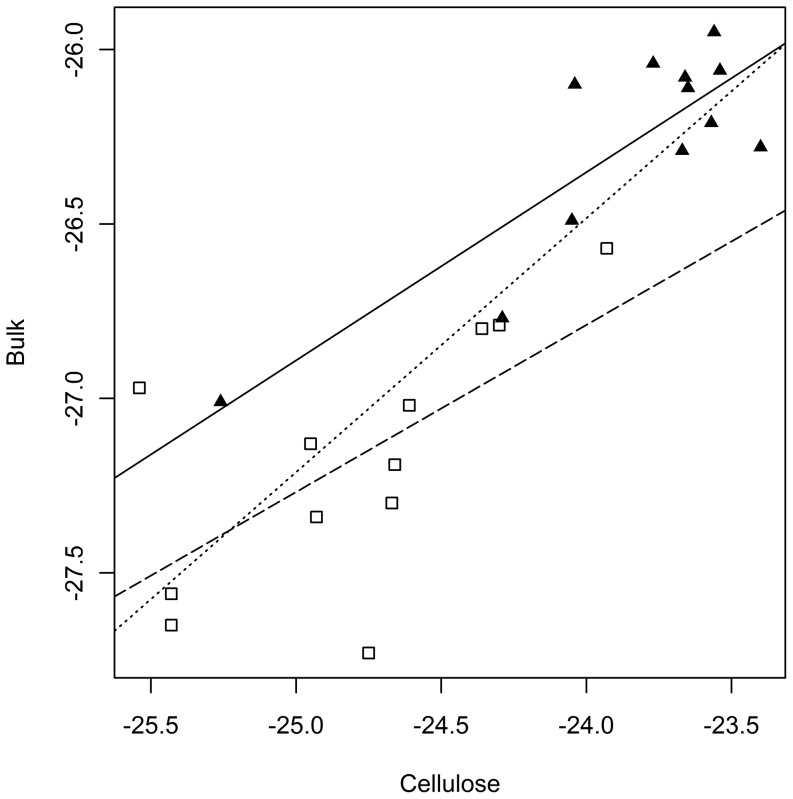
The δ^13^C values of bulk fine root are plotted against cellulose extracts of the fine roots after nine years of irrigation. Irrigated values (open squares) show a reduction of δ^13^C compared to the control (closed triangles). Dotted linear regression shows the overall correlation (*p* < 0.001), the dashed line is the linear regression of the irrigated ratios (*p*  =  0.023) and the solid regression line belongs to the control (*p* < 0.001), (*n*  =  4).

The correlation of the δ^13^C values of cellulose and bulk fine root material showed a strong overall correlation (*p* < 0.001; Figure 4). However, the individual treatments control (*p* < 0.001) and irrigation (*p*  =  0.023) showed a positive δ^13^C values correlation of cellulose and bulk likewise. The obvious treatment effect on the δ^13^C was visible as a shift to a lower δ^13^C ratio of bulk and cellulose (Figure 4, Table 4).

Results of ^14^C analysis performed on roots collected in 2003 estimated fine root ages reflected similar results between the irrigation and the control plots (one-way ANOVA *p*  =  0.674; Table 1). After the nine-year irrigation period, the results showed a significant difference in fine root ages (mixed model *p*  =  0.044). The variation in the data was larger in 2012 than the one in 2003. The mean fine root age was 11.2 yr in 2003 and 7.9 yr in 2012. In 2012, the mean fine root age of the irrigated plots was strongly diminished (5.5 yr) in contrast to the control (10.4 yr).

## Discussion

Water shortage in inner-Alpine valleys force trees to reduce their crowns, shorten their needles, and cause hydraulic failure in extreme drought events [12], [69], [70]. Simultaneously, damage caused by nematodes, insects, fungi, and mistletoes increase because the ability to resist such pests is reduced [71]–[77]. As a consequence, Scots pines die at a higher rate than the average [78]. It has thus been predicted that there will be a shift from sub-boreal Scots pine forests towards the sub-Mediterranean pubescent oak forests in the long-term [79]. However, the alleviation of water shortage by water addition alters the competition situation among all the plants within the Scots pine forest.

### Vegetation shift

After nine years of irrigation, an increase in vegetation cover, mainly due to the better closure of Scots pine crowns, was observed. In contrast, pubescent oak and other drought adapted shrubs such as *Ligustrum vulgare*, *Berberis vulgaris*, and *Arctostaphylos uva-ursi* decreased. Subsequently, with the increase in tree cover, a decrease of the herb (e.g., *Carex alba*) and moss cover was observed, whereas the shrub cover remained unaffected. A change in species richness, however, was not observed, although new species came in and a few species disappeared. Among the new species, some are known to predominantly prefer wet or variably moist conditions, e.g., *Populus tremula*, *Clematis vitalba*, or *Acer* spp. [80], [81]. Species such as *Viola pyrenaica, Trifolium montanum*, *Ligustrum vulgare*, and *Teucrium chamaedris* were naturally present in the control but strongly reduced in the irrigation plots. The reason for this exclusion is most likely caused by fast changes between wet and dry conditions, and competition with better adapted pioneers (e.g. [82]). These effects are limited to annual or herbaceous perennial plants with a short lifespan. Nevertheless, the irrigation also affected, in addition to crown transparency, the mortality of the Scots pine [1], [2]. Rather surprising is the fact that there is a lack of literature using plant indicator values to demonstrate shifts in vegetation upon environmental change, as has been shown earlier for mires or alpine meadows [83], [84]. Undoubtedly, the water availability indicated by moisture variability and moisture values are consistent. However, the assessment of the plant community additionally reveals subtle changes like a decrease in the light value or an increase in nutrient availability. The light value is reduced by the effect of a closing canopy in the irrigated plots, whereas the nutrient availability seems to be triggered directly by the increase in soil moisture content [85]. The decrease in the light value was also detected earlier by Dobbertin et al. [12] having recorded a decrease in crown transparency and an increase in needle length in the irrigated plots.

### Root morphology

**Figure 5:**
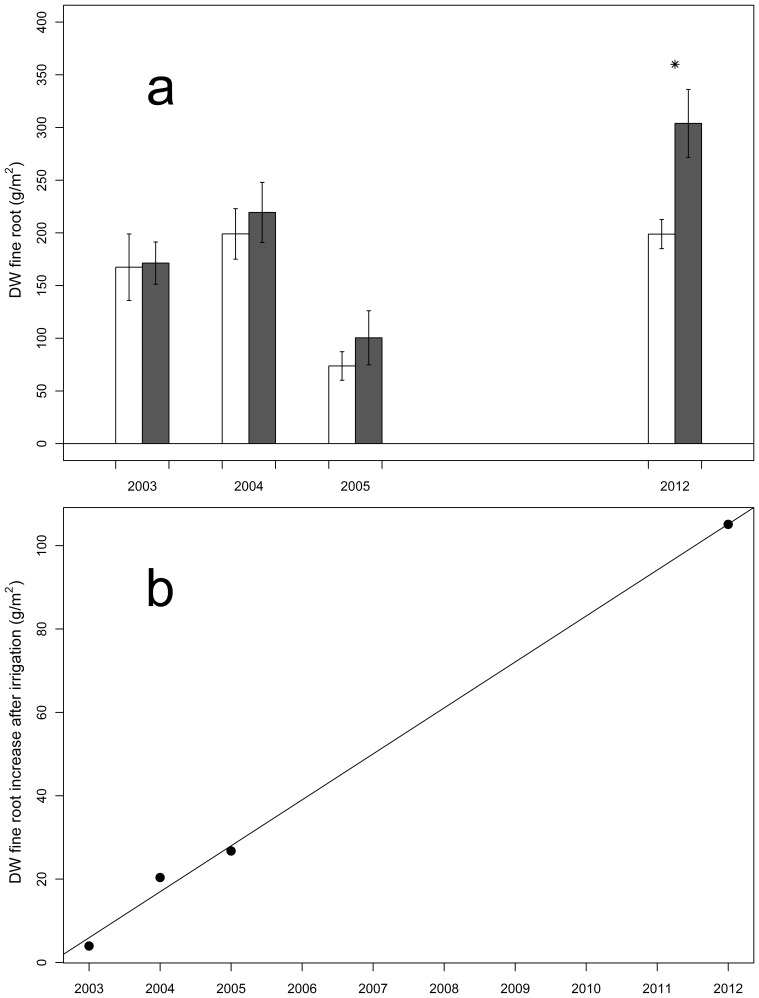
Fine root dry weight compared to data from Brunner et al. [23] (error bars  =  standard error), (*n*  =  4). (a) Fine root dry weight (g/m^2^) are shown as bar plots from 2003, 2004 and 2005 [23] compared to the 2012 detected. *P*-values < 0.05 calculated with a mixed model followed by ANOVA are marked with *. (b) A significant positive linear correlation of the Scots pine fine roots increased means could be shown (R2 =  0.9958, *p*-values =  0.0014).

Nine years after the start of the irrigation, the treatment resulted in a significant increase in the fine root biomass to a near-to-significant increase of the fine root length for *P. sylvestris*. Such a trend was observed as well in hardwood forests in the US [86], [87], indicating that water seems to stimulate root elongation after suffering from long lasting dry periods [88]. The measured decrease in fine root tips, which was significant for pubescent oak and a tendency for Scots pine, appears to be caused by the detected elongation of the fine roots. The total increase in fine root dry weight per soil volume after irrigation is unsurprising (e.g. [89]). Interestingly this change wasn't detected immediately. In the initial years of the irrigation experiment no significant change could be detected [23]. Comparing the results from Brunner et al. [23] with ours evidently demonstrates the slow adaptation capacity of the Scots pine fine root system in the drought afflicted Pfynwald (Fig. 5). Bakker et al. [21] demonstrated a shift of fine roots to shallower soil layers with increasing soil moisture, and consequently, the overall biomass was increased in the top layers (0–10 cm). Moreover, Leuschner et al. [90] found in their driest stand of *Fagus sylvatica* (precipitation < 520 mm yr^−1^), which is equivalent to our study site, the lowest fine root biomass. In a comparable study in a semi-deciduous forest in Panama, similar results were observed with irrigation in a water limited situation, with an increase of the fine root biomass in the upper most soil layers [89]. This drought effect often referred to as *deep rooting strategy* (e.g. [91]) appears to be suspended by the irrigation treatment, resulting in the detected fine root biomass increase.

With an increase of root length and root biomass, a change in the SRL obviously cannot be expected. Indeed, Ostonen et al. [24], in their meta-analysis of SRL, did not record a change in SRL, either due to irrigation or due to drought. While only a few studies have detected changes in SRL mediated by irrigation, they were also combined with fertilization (e.g. [92]). However, SRL is best known for differentiating between species or plant strategies [93] as it is known for leaf and needle traits such as specific leaf area or leaf dry matter content (e.g. [93]–[95]). The comparison of the two dominating tree species in our study revealed large differences, with the pubescent oak having a much higher SRL (2.37±0.23 cm mg^−1^) than the Scots pine (0.73±0.04 cm mg^−1^). Similar values were recorded by Ostonen et al. [24] for *Q. robur* (1.29±1.79 cm mg^−1^) and *P. sylvestris* (0.75±1.05 cm mg^−1^), leading to the conclusion that oaks with 2–3× longer roots per weight unit might have an advantage in water absorbance compared to pines. Overall, this evidence supports the notion that the pubescent oak has greater competitiveness compared to the Scots pine under a drought situation [4]. Hertel et al. [96] recently showed a positive correlation of the RAI to annual precipitation in beech forests. In our study the RAI reacts positively to the increased water availability. These trends indicate a marginal and tardy morphological plasticity of mature pine roots with the same evolutionary background.

In contrast, air warming or drought treatments are known to decrease the root length of *Quercus* sp. [97]–[99]. These results are potentially triggered by an accompanying nutrient deficiency [100], [101], taking into consideration the fact that nutrients and water availability are often highly linked. Due to known drought tolerance following a deep rooting strategy [102], the fine roots of *Q. pubescens* in Pfynwald perhaps neglect the upper soil layer (0–10 cm).

### Root chemical properties

The fine root structure of *P. sylvestris* reacted strongly to the irrigation treatment. On the one hand, the amount of N in bulk roots was reduced even if the irrigation increased the N input slightly. This phenomenon is known and is described in the literature, where the increase in N results in plant growth stimulation (e.g. [103]–[105]). Eilmann et al. [13] clearly demonstrated the increased pine growth at our study site. The N in the tree fine roots is therefore diluted by the boost of fixated C under irrigation. In addition, the priority of biomass accumulation, including N integration, is based on aboveground structures (regenerative organs, needle production, stem growth) [106]. Meanwhile, the increased irrigation in our study potentially enhanced N leaching. These results agree with the assumption of a water limited forest in the Pfynwald rather than N limitation. On the other hand, the isotopic ratio of δ^13^C is strongly influenced by additional water supply. The comparison of fine root bulk with cellulose showed a significant correlation. This indicates, similar to the results of Eilmann et al. [13], that photosynthates were used in a coupled ratio. The shift to overall reduced δ^13^C merely demonstrates an increased photosynthetic activity of Scots pine by irrigation due to the discrimination of the ^13^C isotope [107]. There is no enhanced cellulose production in relation to other C-associated root compounds. The fine root composition of cellulose, lignin, and phenols under irrigation was unchanged. The fine root rough structure was preserved, and revealed a conservative nature similar to King et al. [108] who detected marginal changes with altered nutrient availability. In addition, it is possible that, due to the slow turnover of tree roots, adaptations are still lagging after nine years of irrigation. This is rather surprising, keeping in mind that phenolic compounds [28] or lignin [27] can increase with stress caused by pathogens.

### Radiocarbon

Radiocarbon analysis revealed a decrease in mean fine root age by different treatments of *P. sylvestris* after nine years of irrigation. Up to now, a treatment induced alteration of the mean fine root age has not been demonstrated using this new technique of ^14^C dating. Nonetheless, appropriate caution is needed here. First, the total root age varies considerably depending on the root lifespan assessment method [109], and second, considering the increased fine root biomass with irrigation, it is possible that we have detected more young and newly produced roots in relation to old roots. Black et al. [110] and Chapin et al. [111] observed in their studies that root elongation tends to be negatively correlated with their longevity. Interestingly, water increase as well as decrease can have negative effects on fine root lifespan [112], [113]. However, both studies are barely comparable to our findings; not only was the detection period very short (100 days and 1 year, respectively), but also additional factors such as competition or logging could have had crucial effects on the fine root lifespan [114]. A similar trend, as in our results, was observed by Yuan and Chen [115] and Finér et al. [116], who recorded an increase in the turnover rate caused by an increase in the mean annual precipitation. It also appears that, using the fine root lifespan as a fine root turnover approximation, is risky in a temperate zone due to phenology [114]. In our case, the samples were collected in the same spring period, which, accordingly, reduces the phenological influence. Subsequently, we postulate an increase in fine root turnover rate in the presence of increasing water availability. Furthermore, it is obvious that the ^14^C bomb peak model is more uncertain the more recent the samples are because the bomb peak effect is flattening out due to the ocean uptake and recently fossil fuel combustion [117]. Moreover, the turnover rate of fine roots is a highly discussed topic and represents a major factor for C sequestration which will trigger future climatic conditions [115], [118]. Precipitation and drought, amongst others, are relevant factors which will directly influence future climate and CO_2_ feedback cycles [119], [120].

In conclusion our data imply that the responses belowground to irrigation are less conspicuous than the more rapid adaptations aboveground. Lagged and conservative adaptations of tree roots with decadal lifespans are challenging to detect, hence demanding for long-term surveys. Furthermore, interactions of treatments with biogeochemical processes operate on longer time scales and cannot be detected in short-term studies, which highlights the importance of long-term experiments at natural forest sites (e.g. [121]–[123]). Investigations concerning fine root turnover rate and degradation processes under a changing climate are crucial for a complete understanding of C cycling.

## Supporting Information

Table S1 Translation and specified classification for the indicator values by Landolt (1977).
(DOC)

Table S2 Species list with the mean abundance (%) in the control and irrigated plots. One-way ANOVA: *p* < 0.05  = significant, denoted; *p* ≤ 0.05–0.1  =  non-significant, denoted; *p* > 0.1  =  non-significant (ns).
(DOC)
