# Correction: Nine Years of Irrigation Cause Vegetation and Fine Root Shifts in a Water-Limited Pine Forest

**DOI:** 10.1371/journal.pone.0116642

**Published:** 2014-12-23

**Authors:** The PLOS ONE Staff

There are errors in the Vegetation section of the Results. Please see the corrected paragraph below.

The vegetation type of the pine forest belongs to the *Erico-Pinetum caricetosum albae* Br.-Bl. [68]. The species richness and the coverage rate of vegetation assessment are listed in table 2 and the full plant list and plant species mean abundance is illustrated in table S2 of the supplemental material. Species richness did not differ between control and irrigated plots. The mean number of species in irrigated plots was 41.8, whereas control plots showed a mean number of species of 39.3. Mean vegetation cover as well as the coverage of trees showed a tendency for increase at irrigated sampling sites compared to control plots, although not significantly different. In contrast, the coverage of herbs, mosses, and dead wood showed a decrease at irrigated sampling sites compared to control plots, with significant differences for herbs and dead wood. The shrub cover didn't show any differences between the treatments. The calculated Landolt indicator values were significantly higher for moisture (*p*  =  0.004), moisture variability (*p*  =  0.037) and nutrient value (*p*  =  0.041), but significantly lower for continentality (*p*  =  0.019), and the reaction value (*p*  =  0.032). These five values were significantly explanatory for the NMDS distribution of the sampling plots (Figure 3). Using a NMDS technique, the plots that experienced identical treatment showed significant clustering (PERMANOVA: *p*  =  0.023, Figure 3).

**Table 2:** Vegetation assessment results for mean number of species per plot (species richness) and mean percentage coverage of mean vegetation, dead wood, trees, shrubs, herbs, and mosses after nine years of irrigation and their standard deviation (SD). One-way ANOVA: *p*-values in bold are significant (*p* <0.05), (*n*  =  4).

		Control		Irrigated		*p*
		mean	SD	mean	SD	
Species richness		39.3	1.9	41.8	5.1	0.395
Cover (%)	Mean vegetation	29.3	18.7	50.5	22.2	0.193
	Dead wood	9.5	3.3	4.5	1.9	**0.040**
	Trees	55.0	10.8	70.0	8.2	0.069
	Shrubs	16.3	7.5	16.3	4.8	1.000
	Herbs	28.8	8.5	13.8	7.5	**0.039**
	Mosses	50.8	14.2	39.5	18.9	0.378

There are errors in Table 2. There is a mistake in the statistical analysis of the *p*-values in the table. The authors have provided the corrected table below.
