# Supplementary figures and images for: Nine Years of Irrigation Cause Vegetation and Fine Root Shifts in a Water-Limited Pine Forest
Source: PLoS One. 2014 May 6;9(5):e96321. doi: 10.1371/journal.pone.0096321 (PMC4011741; doi:10.1371/journal.pone.0096321)

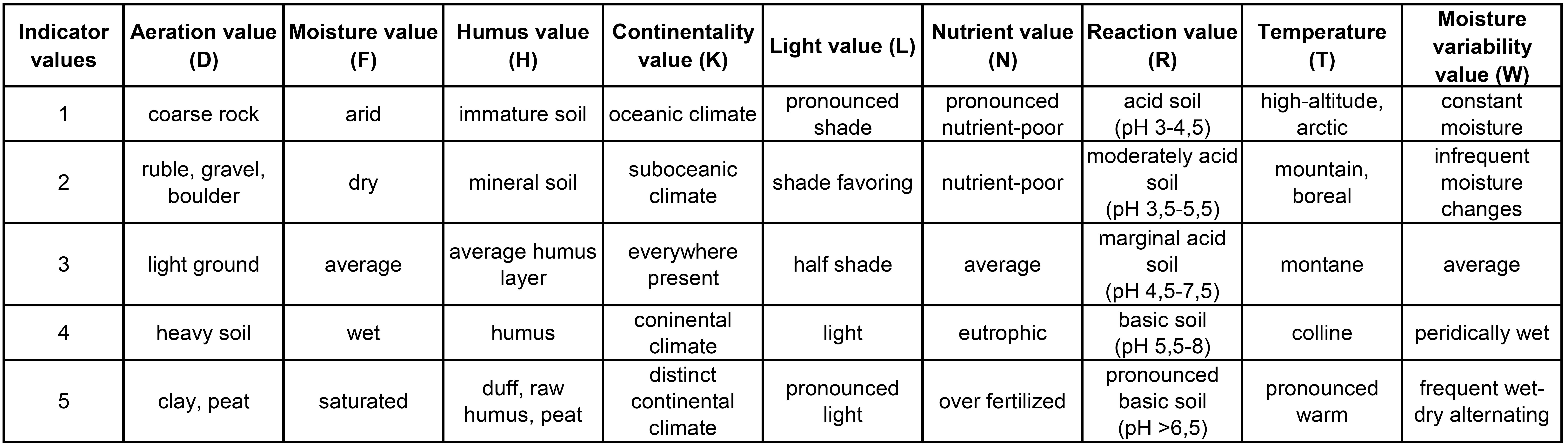

Supplement: Table S1 — Translation and specified classification for the indicator values by Landolt (1977). (DOC) [file pone.0096321.s001.doc]

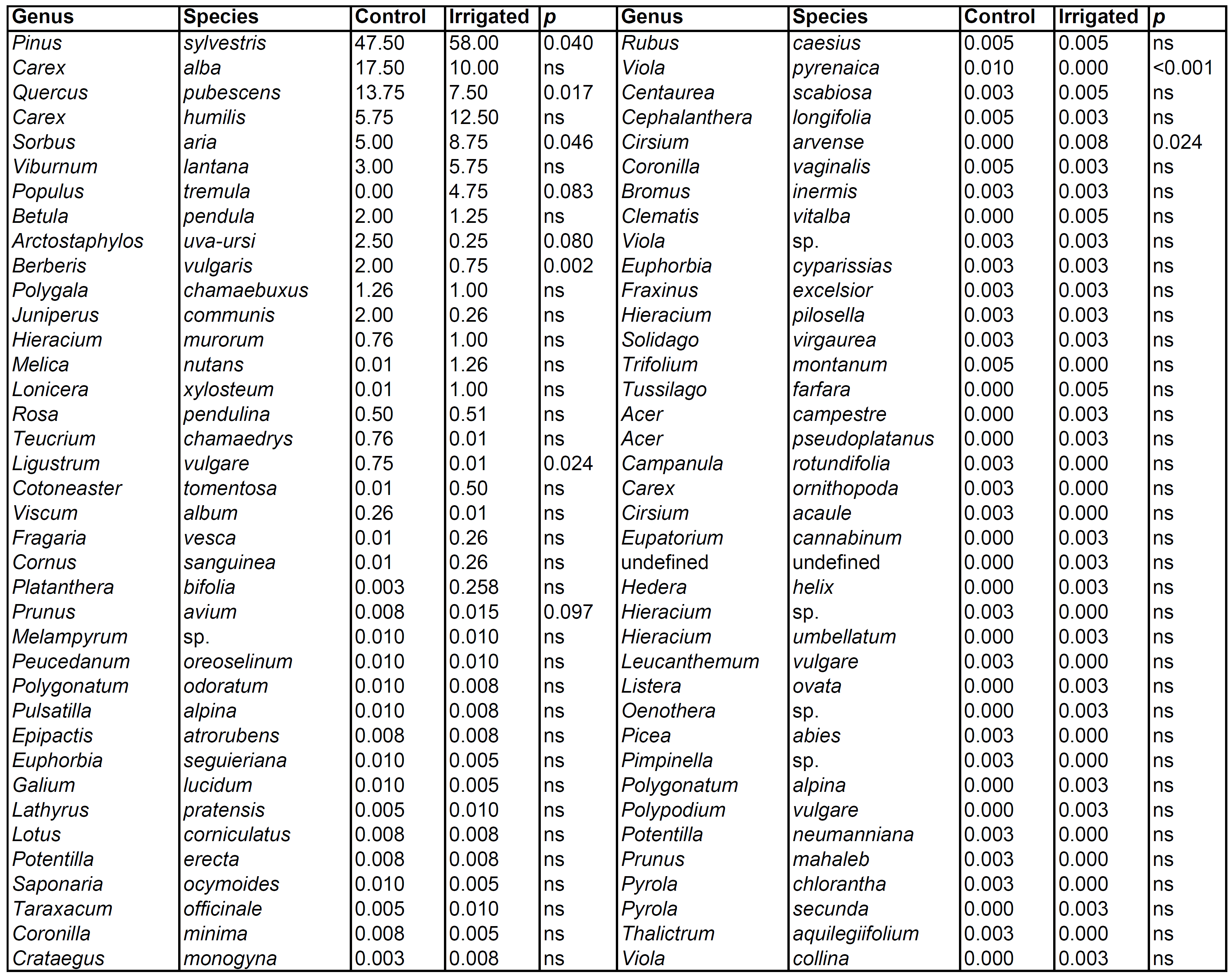

Supplement: Table S2 — Species list with the mean abundance (%) in the control and irrigated plots. One-way ANOVA: p < 0.05 = significant, denoted; p ≤ 0.05–0.1 = non-significant, denoted; p > 0.1 = non-significant (ns). (DOC) [file pone.0096321.s002.doc]
